# Mad River Dental Society

**Published:** 1871-10

**Authors:** 


### MAD RIVER DENTAL SOCIETY.

The semi-annual meeting of this body was held on the 11th and 12th of September at Yellow Springs. There was not a large number of the members present, but all who were ther participated in the proceedings, so that it was a very profitable, interesting and pleasant time. The discussions were good. The clinic by Dr. Blount, illustrating the use of smooth points in filling, was an entire success, showing most conclusively that it is practicable to fill teeth as well and as rapidly with smooth as with serrated pluggers; and in further illustration of this it is worthy of note that both Dr. Blount and Dr. Williams, of Xenia, have used smooth points only, for the last eight months, and they regard their operations made during this time superior to anything they had ever performed before, and they are certainly very good in appearance.

After a meeting of two days, the Society adjourned to meet in Xenia in May next.

This is one of the most active local societies with which it has been our privilege to meet. T.

